# Impact of Food Exposome on Atherosclerotic Plaque Stability: Metabolomic Insights from Human Carotid Endarterectomy Specimen

**DOI:** 10.3390/ijms26147018

**Published:** 2025-07-21

**Authors:** Emilie Doche, Barbara Leclercq, Constance Sulowski, Ellen Magoncia, Catherine Tardivel, Ljubica Svilar, Gabrielle Sarlon-Bartoli, Jean-Charles Martin, Michel Bartoli, Alexandre Rossillon, Laurent Suissa

**Affiliations:** 1 Stroke Unit, University Hospital La Timone, AP-HM, 13005 Marseille, France; 2 Centre de Recherche en Cardiovasculaire et Nutrition, INSERM 1263, INRAE 1260, Aix Marseille University, Campus Timone, 13005 Marseille, France; 3 Medecine Vascular and Hypertension Department, University Hospital La Timone, AP-HM, 13005 Marseille, France; 4 Vascular Surgery Department, University Hospital La Timone, AP-HM, 13005 Marseille, France

**Keywords:** metabolomics, coffee, diet, plant based, niacin, atherosclerosis, carotid stenosis, endarterectomy, carotid

## Abstract

Carotid atherosclerotic stenosis (CAS) is a leading cause of ischemic stroke. Current understanding of plaque vulnerability remains largely confined to histopathological characterization. Consequently, identifying molecular determinants of plaque stability represents a major challenge to advance prevention strategies. Untargeted metabolomic analysis was performed using mass spectrometry coupled to liquid chromatography on carotid plaques removed from patients with CAS undergoing endarterectomy. To identify factors influencing plaque stability, we compared 42 asymptomatic with 30 symptomatic CAS patients. Associations between each annotated metabolite in plaques and asymptomatic CAS status were assessed using logistic regression models. Asymptomatic patients exhibited lower plasmatic levels of C-reactive protein (CRP) and higher HDL-cholesterol. Within the plaques, caffeine and its catabolites, paraxanthine and methylxanthine, were associated with plaque stability and were correlated with HDL-cholesterol. Additional plant-based diet biomarkers including N5-acetylornithine, gentisic acid, proline betaine, and homostachydrine were also associated with plaque stability. In contrast, N-methylpyridone carboxamides, reflecting niacin excess, involved in vascular inflammatory processes, were both associated with plaque vulnerability and also correlated with higher CRP. Our findings provide molecular evidence that plant-based diets, including coffee, promote carotid plaque stability, while excessive niacin intake, linked to processed foods, may be detrimental. Metabolomics offers new insights into food exposome-related vascular risk.

## 1. Introduction

Atherosclerosis is a major global health issue [1], and its increasing burden, particularly among younger populations [2], is largely attributed to vascular risk factors such as tobacco use, poor dietary habits, obesity, sedentary lifestyles, and emerging threats such as air pollution [3] and microplastics [4]. Vascular events are prevented by antiplatelet therapy, anti-inflammatory agents [5], and the control of modifiable risk factors, as emphasized in comprehensive plans such as Life’s Essential 8 [6]. Carotid atherosclerosis is a leading cause of ischemic stroke (IS) and transient ischemic attack (TIA) and more generally a surrogate marker of vascular risk [7].

Stroke risk associated with carotid atherosclerotic stenosis (CAS) is primarily assessed by the degree of luminal narrowing, which currently guides surgical prevention strategies [8]. Although stroke risk increases with degree of stenosis [7], it persists across all carotid atherosclerotic lesions [9]. In situ thrombosis and/or embolic risk resulting from thrombus formation on a vulnerable plaque plays a central role in the pathophysiology of ischemic events. The concept of vulnerable plaque is supported by specific histopathological features assessable on vascular imaging, such as a large lipid core, intraplaque hemorrhage, or ulcerated plaque [10,11]. Despite advances in imaging techniques and histopathological analyses, those current methods remain limited in their ability to capture the full biological complexity of disease progression. Imaging modalities provide valuable structural and anatomical information but fall short in detecting the underlying biochemical and molecular changes that drive plaque vulnerability. These limitations highlight the need for complementary approaches that can reveal the molecular underpinnings of plaque development and vulnerability. Metabolomics, the comprehensive study of small-molecule metabolites, offers a powerful tool to bridge this gap by providing insights into metabolic alterations associated with atherosclerosis. Currently, we have few data linking plaque stability to its molecular composition in humans [12,13,14].

Advancing our understanding of the determinants of plaque stability at the molecular level remains a critical challenge for improving preventive strategies in carotid atherosclerotic disease and reducing stroke risk. Untargeted metabolomics represents a powerful approach to elucidate metabolic pathways involved in disease and to identify novel biomarkers for cardiovascular risk assessment. Here, we applied this omics strategy to human carotid atherosclerotic plaque obtained via endarterectomy. To identify metabolites associated with carotid plaque stability, we compared the metabolome of asymptomatic plaque to that of symptomatic plaque.

## 2. Results

### 2.1. Patient and Plaque Characteristics Associated with Plaque Stability

We analyzed data for 72 patients with CAS who underwent carotid endarterectomy (CEA); characteristics are presented in Table 1. A total of 42 patients (median age 70 years [interquartile range 64.0–77.5], 73.8% men) with asymptomatic CAS (median NASCET 77.5% [70.0–90.0]) were compared to 30 (median age 69.0 years [63.3–76.8], 83.3% men) with symptomatic CAS (median NASCET 77.5% [70.0–90.0]). For symptomatic patients (27/30 [90%] IS, 3/30 [10%] TIA), time between symptoms and CEA was 12 days (8.3–15.8). Asymptomatic and symptomatic patients did not differ in classical risk factors, except for renal function (median eGFR 63.7 mL/min/m^2^ [51.8–79.3] vs. 91.1 [76.1–98.9], *p* < 0.001). Asymptomatic patients more often received antiplatelets (85.7% vs. 56.7%, *p* = 0.006) and lipid-lowering therapies (76.2% vs. 36.7%, *p* < 0.001) as a basic home treatment. Plasma C-reactive protein (CRP, detection threshold: 0.5 mg/L) was significantly lower in asymptomatic than symptomatic patients (1.75 [0.73–4.65] vs. 3.90 [2.03–7.65], *p* = 0.022), and high-density lipoprotein cholesterol (HDLc, g/L) was higher (0.46 [0.38–0.57] vs. 0.39 [0.35–0.43], *p* = 0.010). As a biomarker of cardiovascular disease [15], the CRP/HDLc ratio was significantly lower for asymptomatic than symptomatic patients (4.00 [1.43–10.1] vs. 10.5 [5.03–19.5], *p* = 0.004). Low-density lipoprotein cholesterol (LDLc, g/L) was lower in asymptomatic patients (0.72 [0.50–0.91] vs. 0.86 [0.66–1.16], *p* = 0.090). According to masked macroscopic descriptions, stable plaque was more often calcified (35.9% vs. 11.5%, *p* = 0.029) and less ulcerated (5.13% vs. 34.6%, *p* = 0.002) than unstable plaque.

### 2.2. Molecular Characteristics of Stability in Carotid Atherosclerotic Plaque

**Figure 1:**
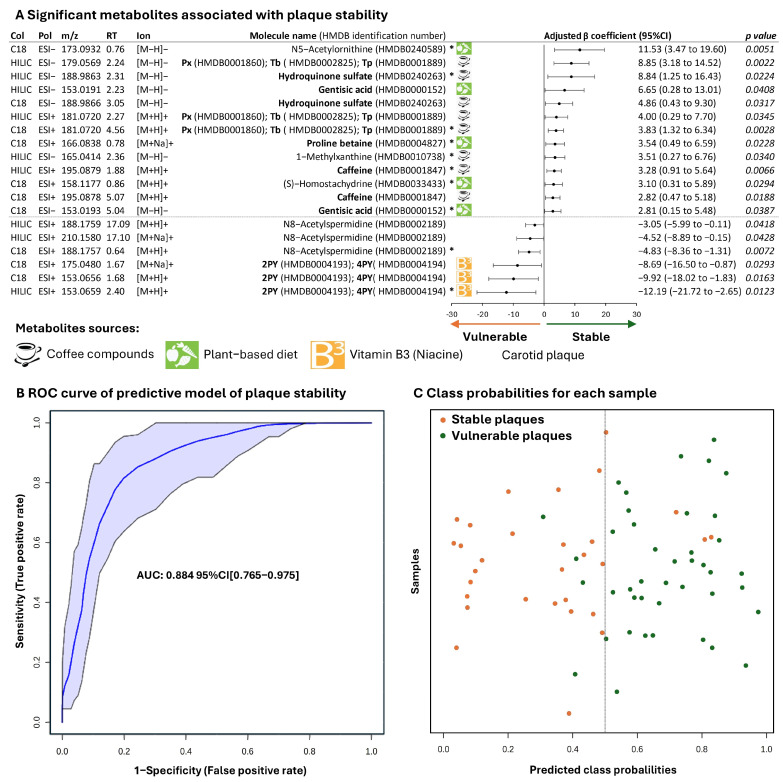
Molecular characteristics of stability in carotid atherosclerotic plaque. Significant metabolites associated with plaque stability are presented with their adjusted β-coefficient. (**A**) A predictive model of plaque stability was built using the most significant adduct (*) of each significant metabolite. Predictive properties of the model are presented with a receiver operating characteristic (ROC) curve (**B**) and class probability of each sample (**C**). Abbreviations: CAS: carotid artery stenosis, Col: high performance-liquid chromatography column used: C18 or HILIC: hydrophilic interaction liquid chromatography, ESI: electrospray ionization, HMDB: Human Metabolome Database, Pol: polarity, 2PY: N-methyl-2-pyridone-5-carboxamide, 4PY: N1-methyl-4-pyridone-3-carboxamide, Px: paraxanthine, RT: retention time, Tb: theobromine, Tp: theophylline, AUC: area under the ROC curve.

The metabolome of each carotid atherosclerotic plaque, established in two different chromatographic conditions (C18 and HILIC) by LC-MS, is presented in Supplementary Data (Dataset S1). A total of 727 features (corresponding to 389 metabolites) were identified with high confidence [16] in at least 90% of samples. Nineteen features (for annotation details, see Supplementary Figure S2) corresponding to 10 metabolites were significantly associated with plaque stability (Figure 1A). The most significant adduct of each metabolite was used in the predictive model of plaque stability (linear SVM). The area under ROC curve, smoothed after 100 cross-validations (Figure 1B), was 0.884 (95% CI 0.765–0.975). The predicted class probabilities provided by this model are presented in Figure 1C. Only 8/72 (11.1%) samples were misclassified, with four false positives and four false negatives. Ten metabolites were related to plaque stability (Figure 1A), and we grouped them according to their endogenous or exogenous source and/or biological pathway. Coffee consumption [17,18,19,20] and plant-based diet [20,21,22,23] biomarkers were associated with plaque stability, and niacin [24] and polyamine catabolites [25] with plaque vulnerability.

### 2.3. Metabolites Associated with Plaque Stability

**Figure 2:**
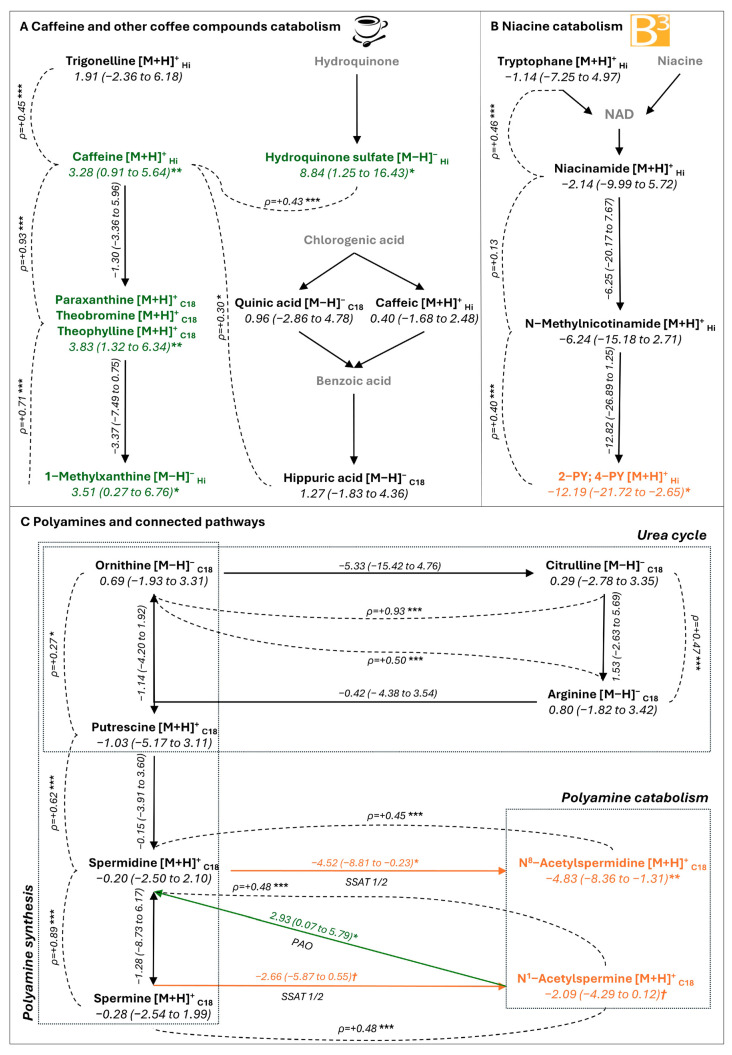
Significant metabolites in their biological pathways: caffeine and other coffee compound catabolism (**A**), niacine catabolism (**B**), and polyamines and connected pathways (**C**). Metabolites are presented with adjusted β-coefficients (in green: significantly associated with plaque stability, in orange: significantly associated with plaque vulnerability, in grey: metabolites not found). ➝ Biological pathway, --- Spearman correlation (ρ-coefficient). SSAT: spermidine/spermine n-acetyltransferase, PAO: polyamine oxydase *** *p* < 0.001, ** *p* < 0.01, * *p* < 0.05, † *p* < 0.1.

Caffeine (*aβ*: 3.28 [95% CI 0.91 to 5.64]) and its catabolites paraxanthine (*aβ*: 3.83 [1.32 to 6.34]) and methylxanthine (*aβ*: 3.51 [0.27 to 6.76]) were significantly associated with plaque stability. Levels of caffeine and its catabolites were strongly positively correlated (Figure 2A). Theobromine and theophylline, direct catabolites of caffeine, were not distinguishable from paraxanthine under both chromatographic conditions used for untargeted metabolomics. Moreover, the content of other known coffee components was significantly correlated with caffeine content; examples are trigonelline (ρ = +0.45), hippuric acid (ρ = +0.30), and hydroquinone sulfate (ρ = +0.43), a derivative of hydroquinone. Hydroquinone sulfate, as for caffeine and its catabolites, was significantly associated with plaque stability (*aβ*: 8.84 [1.25 to 16.43]). Other metabolites from fruits and vegetables significantly associated with plaque stability were N5-acethylornithine (*aβ*: 11.53 [3.47 to 19.60]), gentisic acid (*aβ*: 2.81 [0.15 to 5.48]), proline betaine (*aβ*: 3.53 [0.49 to 6.59]), and homostachydrine (*aβ*: 3.10 [0.31 to 5.89]).

Hence, biomarkers indicative of coffee consumption and a plant-based diet were detected within carotid atherosclerotic plaque and were associated with plaque stability.

### 2.4. Metabolites Associated with Plaque Vulnerability

We found terminal metabolites of niacin (B3 vitamin), N-methyl-2-pyridone-5-carboxamide (2PY), and N1-methyl-4-pyridone-3-carboxamide (4PY) in plaque, but these were not separable under both chromatographic conditions. 2PY/4PY were negatively associated with plaque stability (*aβ*: −12.19 [−21.72 to −2.65]) (Figure 1A). According to the niacin catabolism pathway, 2PY/4PY content was strongly correlated with the content of their precursor, N-methylnicotinamide (ρ = +0.40) (Figure 2B).

Acetylated forms of polyamines, N8-acetylspermidine (*aβ*: −4.83 [−8.36 to −1.31]) and N1-acetylspermine (*aβ*: −2.09 [−4.29 to 0.12], *p* = 0.064), were negatively associated with plaque stability (Figure 1A). All the pathways of polyamines (synthesis from urea cycle to catabolism) were reconstituted, with strong correlation between metabolite levels at every step (Figure 2C). Although polyamines (putrescine, spermidine, and spermine) were not linked to plaque stability, the ratios of N8-acetyspermidine/spermidine and N1-acetylspermine/spermine, expressing spermidine/spermine N-acetyltransferase (SSAT 1/2) activity, were negatively associated with plaque stability (*aβ*: −4.52 [−8.81 to −0.23] and *aβ*: −2.66 [−5.87 to 0.55], *p* = 0.058). Conversely, the spermidine/N1-acetylspermine ratio, expressing polyamine oxydase (PAO) activity, was significantly associated with asymptomatic plaque (*aβ*: 2.93 [0.07 to 5.79]). So, catabolism of polyamines but not polyamines per se was related to plaque vulnerability.

### 2.5. Associations Between Metabolites Inside Plaque, Systemic Inflammation, and Lipid Profile

**Figure 3:**
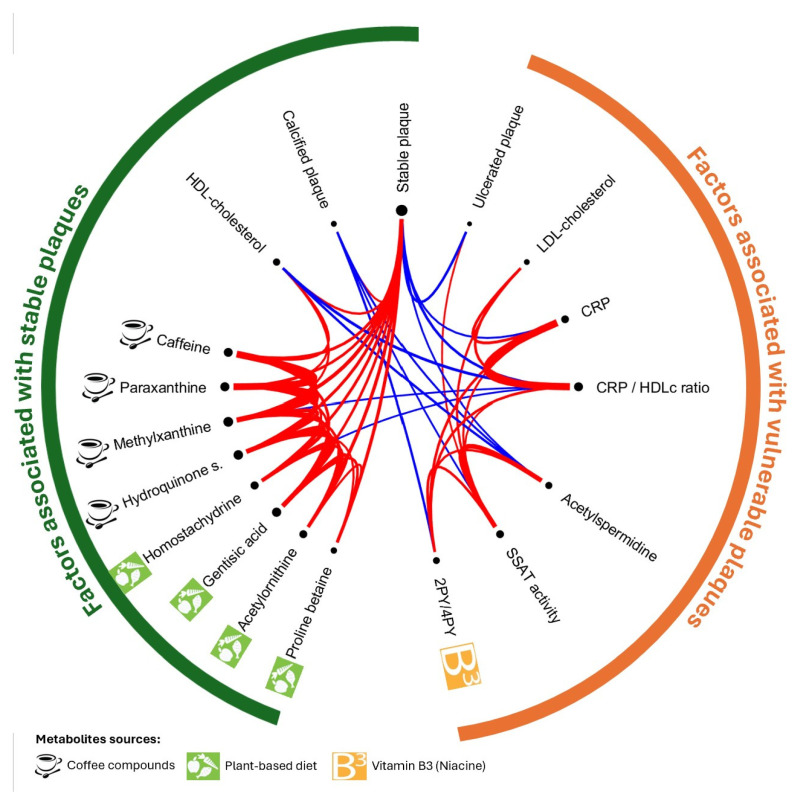
Schemaball representing Spearman correlation matrix of all significant parameters associated with stable plaque. In red: positive correlation, in blue: negative correlation, line thickness reflecting strength of correlation. 2PY/4PY: N-methyl-2-pyridone-5-carboxamide/N1-methyl-4-pyridone-3-carboxamide, SSAT (spermidine/spermine-N1-acetyltransferase) activity assessed by acetylspermidine/spermidine ratio.

Because CRP, HDLc, and LDLc were related to plaque stability, we analyzed Spearman correlations between these plasmatic variables and significant metabolites inside atherosclerotic plaque in Figure 3 (network diagram in Supplementary Figure S3 and correlation matrix in Supplementary Table S1). Asymptomatic plaque was significantly correlated with metabolite biomarkers of coffee consumption (median ρ = +0.40) and plant-based diet (median ρ = +0.41), HDLc level (ρ = +0.32), and the calcified aspect (ρ = +0.27). Conversely, it was negatively correlated with CRP level (ρ = −0.27) (and CRP/HDLc ratio, ρ = −0.36), N8-acetylspermidine level (ρ = −0.25), and ulcerated plaque (ρ = −0.35). Coffee consumption biomarkers were correlated with plant-based diet biomarkers, and HDLc level (median ρ = +0.28). Systemic inflammation, assessed by CRP level, was correlated with SSAT activity (acetylspermidine/spermidine ratio, ρ = +0.28), niacin catabolites (2PY/4PY, ρ = +0.35), and LDLc level (ρ = +0.38). Ulcerated plaque was positively correlated with SSAT activity (ρ = +0.24), whereas calcified plaque was inversely correlated with acetylspermidine level (ρ = −0.28), SSAT activity (ρ = −0.25), and niacin catabolites (ρ = −0.32).

So, stable plaque, with calcification, was related to plant-based diet and coffee biomarkers in plaque, which were themselves correlated with HDLc level. Vulnerable plaque, characterized by more ulceration, was related to systemic inflammation and with intraplaque niacin catabolites and SSAT activity, both related to CRP level.

### 2.6. Metabolites of Mediterranean Diet and Plaque Stability

**Figure 4:**
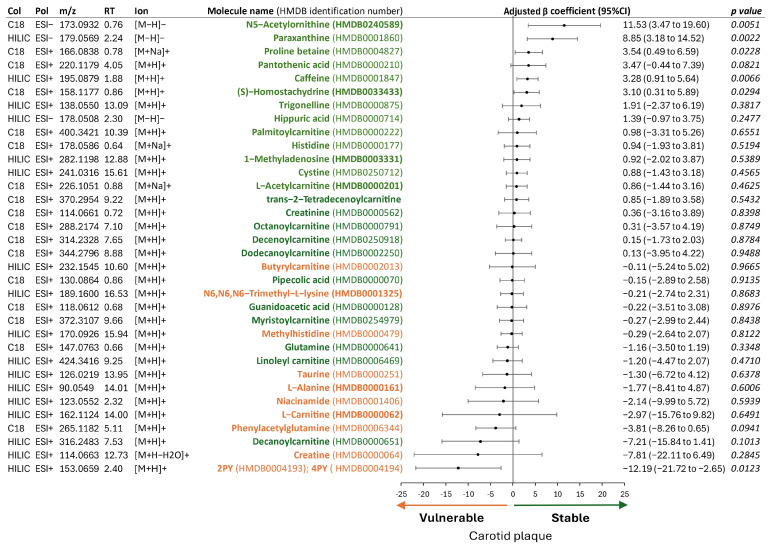
Metabolites from the Mediterranean diet and their association with plaque stability. List of metabolites increased (in green) or decreased (in orange) in level in plasma with a Mediterranean diet, according to Smith et al. [22]. Metabolites found inside plaque are presented with their adjusted β-coefficient, listed in descending order. Abbreviations: Col: high performance-liquid chromatography column used: C18 or HILIC: hydrophilic interaction liquid chromatography, ESI: electrospray ionization, HMDB: Human Metabolome DataBase, Pol: polarity, 2PY: N-methyl-2-pyridone-5-carboxamide, 4PY: N1-methyl-4-pyridone-3-carboxamide, RT: retention time.

Because most of the significant metabolites were biomarkers of a plant-based diet, we extracted from the metabolome of each plaque the metabolites from the metabolomic signature of the Mediterranean diet proposed by Smith et al. [22]. The association of these selected metabolites with plaque stability is reported in Figure 4.

Inside the plaque, metabolites with increased plasma level with a Mediterranean diet [22] were mainly associated with plaque stability. Conversely, metabolites with decreased plasma level with a Mediterranean diet [22] were mainly associated with plaque vulnerability. So, plasma metabolites of the Mediterranean diet were found in plaque and influenced its stability.

## 3. Discussion

For the first time and unexpectedly, this untargeted metabolomic study confirmed at a molecular level the major impact of the food exposome on atherosclerosis carotid plaque, already highlighted by interventional and observational studies on dietary habits and cardiovascular risk [26,27,28,29,30,31,32,33,34]. Although in clinical routine practice, carotid plaque risk is mainly assessed by radiological characterization, little is known about molecular factors of its stability. This study highlighted the metabolites associated with plaque stability or vulnerability, particularly coffee, a plant-based diet, and vitamin B3 (niacin), that originate from diet.

This study supports that coffee consumption has a protective role in atherosclerotic carotid plaque. Indeed, caffeine and all its catabolites were significantly associated with plaque stability. Because the content of other known coffee components was significantly correlated with caffeine content (Figure 2A), caffeine in plaque may be mainly due to coffee consumption. Considerable controversy exists regarding the effect of long-term coffee consumption on vascular risk, but recent findings, based on large observational cohorts, suggest that moderate coffee consumption may lower this risk [26,28,29,30]. In addition to epidemiological studies based on reported coffee consumption, our metabolomic analysis of atheromatous plaque supports this idea, highlighting the association between individual coffee exposure and plaque stability. The mechanisms of action of coffee lowering the risk of plaque instability are not elucidated. Some authors suggest that the beneficial effect may be pleiotropic, depending on several coffee compounds associated with different direct and/or indirect protective mechanisms [18]. In this study, despite different components of coffee found in plaque, only caffeine, its active catabolites, and hydroquinone sulfate were associated with plaque stability, mainly targeting these molecules as potentially responsible for a beneficial effect. However, we cannot exclude that other components of coffee may be responsible for the effect, among the metabolites found or not in this exploratory study using untargeted metabolomic analysis. The underlying mechanisms responsible for the beneficial effect could be considered local but also systemic. In fact, the presence of these metabolites could reflect exposure to chronic coffee consumption causing a systemic and therefore indirect protective effect on plaque.

The protective effects of coffee are thought to involve several mechanisms, including its antioxidant and anti-inflammatory properties and its regulatory effects on carbohydrate and lipid metabolism, as well as vascular tone [18,35]. Coffee components share different pleiotropic properties that are difficult to attribute to one specific metabolite. Phytochemicals from coffee (and plants) enhance the cytoprotective response, including anti-oxidation, detoxification, and anti-inflammation, principally via the activation of the Nrf2 pathway [36]. Specifically, different components of coffee (caffeine, trigonelline, chloro-genic acids) have antioxidant properties by increasing glutathione peroxidase and superoxide dismutase activities and decreasing reactive oxygen species, helping to preserve endothelial function [18,35]. Besides the well-known psychoactive effect, caffeine’s adenosine receptor antagonism property also plays a complex role in inflammation [37]. Although the effect of coffee consumption on CRP level remains controversial [38], we did not find a significant correlation between coffee metabolite levels and CRP level. Of note, the levels of all metabolites of coffee consumption in carotid plaque were positively correlated with plasma HDLc level, a recognized vascular protective factor significantly associated in our study with plaque stability. Consistent with the literature, coffee has been shown to increase plasma HDLc level [18,35,39,40]. Therefore, in line with recent observational studies [26,28,29], based on molecular data in humans, we support the beneficial role of coffee in promoting carotid plaque stability. Among the proposed underlying mechanisms, this study highlights a potential indirect effect of coffee via regulating HDLc level.

In addition to coffee compounds, fruit and vegetable biomarkers [21,23] associated with stable plaque included N5-acethylornithine, gentisic acid, proline betaine, and homostachydrine. Worldwide, multiple interventional and observational studies, mainly based on questionnaires, have demonstrated the major benefit of an alimentary diet [27,31,32,33] for cardiovascular risk prevention, leading to statements and public health programs [6]. Here, metabolites in plaques significantly associated with plaque protection were mainly from fruit consumption [23] or a healthy diet [21,41]. As for coffee, the presence of these metabolites in plaque may reflect dietary habits that contribute to systemic benefits and/or play a direct role in plaque stability. Indeed, several of these metabolites have beneficial effects. For example, gentisic acid, a phenolic compound present in plant-based foods, has anti-inflammatory and antioxidant properties [42,43,44].

Finally, given that levels of carotid plaque-stabilizing metabolites were all positively correlated, their dietary source may be associated with a consistent dietary pattern that favors plant-based foods, akin to the Mediterranean [32,34], plant-based, or Dietary Approaches to Stop Hypertension (DASH) diets [33].

Both inflammation and an abnormal lipid profile constitute fundamental pillars in the pathophysiology of atherosclerosis. Consistent with this notion, we found that inflammation, assessed by plasma CRP level, as well as elevated LDLc level and reduced HDLc level, was associated with plaque instability. As expected, CRP/HDLc ratio, a recognized inflammation–lipid composite marker [45], was increased in symptomatic CAS patients. In carotid plaque, the content of vitamin B3 (niacin) and polyamine catabolites was negatively associated with stable plaque. We discuss their potential role as markers or contributors to local inflammation within plaque.

Levels of metabolites from the polyamine catabolism pathway, such as N8-acetylspermidine, were elevated in unstable plaque. Given that SSAT catalyzes the formation of acetylated spermidine, its activity was inferred by evaluating the ratio of the acetylated to non-acetylated spermidine form. Because the acetylation of spermidine to N8-acetylspermidine is an irreversible reaction [25], this ratio may be a reliable indicator of SSAT enzyme activity. This elevated activity found in unstable plaque may be triggered by polyamine excess or inflammation [46]. So, in the absence of polyamine accumulation in unstable plaque and given the correlation between SSAT activity and plasma CRP level, SSAT activity may be a biomarker of only local inflammatory activity independent of polyamine roles. Indeed, polyamines are potentially beneficial in atherosclerosis [25], but we found no changes in the tightly regulated polyamine synthesis pathway related to plaque stability, including any depletion resulting from increased catabolism. Of note, levels of 2PY and 4PY, which are linked to unstable plaque, were correlated with levels of both systemic and local inflammation biomarkers, including plasma CRP level and SSAT activity. Niacin, an essential diet-derived vitamin, is generally considered beneficial for health, but excessive intake is associated with increased vascular risk. Indeed, as beautifully demonstrated recently by Ferrell et al. [24], 2PY and 4PY, terminal metabolites reflecting excess niacin, promote vascular inflammation and contribute to cardiovascular risk. To prevent pellagra, food staples, particularly flour, have been increasingly enriched with niacin. The growing trend toward a Western diet leads to daily niacin intake close to the tolerable upper level, overflowing the niacin pool and generating PYs [24]. Furthermore, SSAT activity and PY level, assumed as a marker and mediator of local inflammation, respectively, were negatively associated with plaque calcification, features typically linked to reduced inflammatory burden [47].

Diets aiming to reduce cardiovascular risk (Mediterranean [32,34], plant-based, or DASH diets [33]) share common features including increased consumption of fruits, vegetables, and legumes and reduced intake of meat and processed (or ultra-processed) foods. Because we highlighted the effect of the food exposome on carotid plaque stability at the molecular level, we aimed to further explore the dietary origin of these metabolites, with a particular focus on the Mediterranean diet. To this end, we applied the metabolomic signature proposed by Smith et al. [21,22], which was derived from the analysis of plasma metabolite changes in patients after one week of a Mediterranean diet. Of note, levels of metabolites increased by the Mediterranean diet [21,22] were protective in plaque, whereas levels of those decreased by the diet were associated with plaque vulnerability (Figure 4). These findings raise questions about the complex mechanisms underlying the health benefits of healthy diets, which remain incompletely understood. The Mediterranean diet emphasizes fruits, vegetables, legumes, whole grains, and olive oil, primarily via home-cooked meals. Its vascular protective effects are thought to result from combined mechanisms, including improvements in lipid metabolism, antioxidant and anti-inflammatory actions [48,49], enhanced intake of low-glycemic index carbohydrates, increased dietary fiber, and beneficial modulation of the gut microbiota [50]. Moreover, a key advantage of the Mediterranean diet is its focus on home-prepared meals, thereby limiting the intake of processed and ultra-processed foods [51]. The lower consumption of such products, commonly enriched with niacin, may contribute to the reduced levels of PYs observed in the metabolomic profile associated with this dietary pattern [22]. In this context, the PYs identified in our study as potential mediators of plaque instability may reflect dietary exposure to niacin-rich processed foods, as Ferrell et al. [24] previously hypothesized. Thus, these metabolomics findings support current recommendations and public health initiatives, such as Life’s Essential 8 [6], which not only promote individual health but also contribute to environmental sustainability [52].

### Strengths and Limitations

To our knowledge, this is the first study to provide evidence of the biological impact of the food exposome on carotid plaque stability in humans. This cross-sectional observational study is preliminary and exploratory and therefore has several biases and does not allow for establishing causality. Because we analyzed carotid plaque extracted by endarterectomy, our results concern surgical patients and might not represent all CAS patients. We did not include self-questionnaires in the study design, as key findings emerged unexpectedly from an untargeted metabolomic analysis. So, the impact of potential confounders such as socio-economic status or lifestyle could not be assessed. In the absence of prior literature on this topic, we cannot associate the identified food-related metabolites with the duration or intensity of dietary exposure, nor the metabolite half-life. Although previous studies have shown that short-term adherence to a Mediterranean diet can significantly modify plasma metabolomic profiles [22], the molecular changes observed within carotid plaque are more likely to reflect chronic dietary influences. Indeed, long-term adherence to the Mediterranean diet is beneficial for carotid artery atherosclerosis [49]. Finally, possible confounding from other exposures such as medication use could remain despite statistical adjustments. For example, gentisic acid, whose main sources are plants, herbs, spice, fruits, and nuts, is also a minor catabolite of aspirin. Nevertheless, no correlation could be found between gentisic acid level measured in carotid plaque and levels of other catabolites of aspirin nor regular use of aspirin.

Our findings support the relevance of plaque metabolomics as a valuable tool for advancing our understanding of vascular risk factors. Applied to blood samples, metabolomics could become a useful tool to evaluate dietary interventions in clinical studies or adherence to lifestyle guidelines such as the Essential 8 [6] in clinical practice. Other spectrometric approaches have also proven insightful, including recent studies highlighting the vascular risk of micro- and nanoplastics in carotid plaque using pyrolysis–gas chromatography–mass spectrometry [4].

## 4. Materials and Methods

### 4.1. Population and Clinico-Biological Data Collection

In this observational study, conducted in accordance with STROBE guidelines, we included 80 patients with CAS who underwent carotid endarterectomy (CEA) according to current guidelines [8], with metabolomic analysis of the removed plaque, between May 2021 and August 2022 in the vascular surgery department of Marseille University Hospital. Patients who did not report any medical history of IS/TIA in the last 6 months before surgery were considered asymptomatic, and their carotid plaques were classified as stable. Because our aim was to compare stable plaque (no recent cerebrovascular events) and vulnerable/high-risk plaque (recently symptomatic), we chose to exclude patients with a history of ischemic stroke (IS) or transient ischemic attack (TIA) occurring between 1 and 6 months before surgery (*n* = 8) because their plaques may have undergone partial healing during that time, potentially reducing their vulnerability. Symptomatic CAS was con-firmed by stroke physicians (E.D., L.S.) if patients had experienced an IS or TIA in the territory of the culprit artery within one month prior to surgery, with an otherwise negative exhaustive etiological assessment. Medical history and biological data (collected before surgery) were retrospectively extracted from patients’ records. The institutional review board of Assistance Publique-Hôpitaux de Marseille approved this study (No. CSE24-4). No written consent was required.

### 4.2. Collection and Preparation of Carotid Plaque for Metabolomic Analysis

Carotid plaque was collected by a vascular surgeon (M.B., A.R.) during CEA. Eversion endarterectomy was performed for asymptomatic CAS and longitudinal arteriotomy using a shunt, followed by patch closure, for symptomatic CAS. The removed surgical plaque was immediately frozen at −20 °C then transferred to −80 °C until metabolomic analyses in a single batch. At the time of thawing, after a brief ice-cold saline solution (NaCl 0.9%) rinse, each piece was photographed (Supplementary Data Figure S1) and macroscopically described by a single examiner (B.L.) blinded to the symptomatic or asymptomatic status of the patient. Then, an area of interest, corresponding to the region most at risk of causing an ischemic event, according to the macroscopic appearance of the plaque (highest degree of stenosis, ulceration or intraplaque hemorrhage) was cut out for metabolomic analysis (B.L.). Each sample was incubated for 20 min at 4 °C in lysis buffer (7 M urea, 2 M thiourea, 4% dodecyl maltoside, and 50 mmol/L dithiothreitol (Thermo Fisher Scientific, Villebon sur Yvette, France) to denature the fibrin network. Chemical solubilization was completed by manual mechanical grinding with a homogenizing pestle. After centrifugation of samples (13,000× *g*, 15 min, 4 °C), the hydrophilic supernatant was collected and stored at −80 °C until metabolomic analysis. A protein assay with the Bradford colorimetric technique (Thermo Fisher Scientific) was applied to analyze each solubilized sample to standardize the quantity of material used for metabolomic analysis (B.L., C.S., E.M.).

### 4.3. Untargeted Metabolomic Analysis of Carotid Plaque

The metabolome of each carotid plaque was established by liquid chromatography coupled with tandem mass spectrometry (LC-MS) from a volume of hydrophilic supernatant equivalent to 200 μg protein. Metabolites were extracted in solvent by mixing the supernatant into five volumes of methanol. After overnight incubation at −20 °C, samples were centrifuged (13,000× *g*, 15 min at +4 °C) and supernatant was dried by using a nitrogen evaporator before resuspension. The resulting samples were analyzed by using an ESI-Q Exactive Plus mass spectrometer (Thermo Fisher Scientific). Liquid chromatographic separation involved using the DIONEX UltiMate 3000 HPLC system coupled to two different columns to enrich metabolome coverage.

For reverse-phase chromatography, samples were resuspended in 125 μL of a 90:10 Mili-Q water/acetonitrile (hypergrade for LC-MS, LiChrosolv, Supelco, Merck, Molsheim, France) mixture with 0.1% formic acid (Optima LC/MS, Fisher Chemical, Thermo Fisher Scientific) before centrifugation (11,000 rpm, 15 min at +4 °C). A Hypersil GOLD C18 100 × 2.1 mm × 1.9 µm (Thermo Fisher Scientific, France) was used with a mobile phase consisting of 0.1% formic acid in Mili-Q water (A) and 0.1% formic acid in acetonitrile (B) at a flow rate of 0.4 mL/min (40 °C) over 16 min. The gradient started at 0% (B) for 1 min, increased to 100% (B) over 10 min, was maintained at 100% (B) for 2 min, and then returned to 0% (B) in 1 min, and the column was equilibrated at 0% (B) for 2 min. Full scan–ddMS^2^ spectrometric acquisition was applied in positive and negative ionization mode in the *m*/*z* 80–1000 range with a mass resolving power of 35,000 FWHM.

For hydrophilic interaction liquid chromatography (HILIC), samples were resuspended in 125 μL of a 90:10 Acetonitrile/Mili-Q water mixture with 0.1% formic acid before centrifugation. A SeQuant ZIC-HILIC Peek Coated (150 × 2.1 mm × 5 µm) (Merck Millipore, Billerica, MA, USA) column was used with a mobile phase consisting of Mili-Q water plus 16 mM ammonium formate (A) and 0.1% formic acid in acetonitrile (B) at a flow rate of 0.4 mL/min (40 °C) over 27 min. The gradient started at 97% (B) for 2 min, decreased to 70% (B) over 8 min and to 10% (B) in 5 min, then was maintained at 10% (B) for 2 min, was returned to 97% (B) in 1 min and held at 97% (B) until the end of the gradient for column equilibration. Full scan spectrometric acquisition was applied using alternating ionization mode in the 80–1000 *m*/*z* range with a mass resolving power of 35,000 FWHM. Full scan–ddMS^2^ spectrometric acquisition in positive and negative ionization mode was performed on pooled samples for annotation.

Samples were randomly assigned to the injection (5 µL), and quality control samples (QC, a pool of all samples of a biological matrix) were injected several times at the beginning for column equilibration and interspersed every set of five samples throughout the experimental sequence to evaluate the data quality. The extraction blank of the solvent mixture was analyzed at the beginning of the experimental batch sequence. The metabolomics data have been deposited in the MetaboLights repository with the study identifier MTBLS12303 (https://www.ebi.ac.uk/metabolights/MTBLS12303; accessed on 6 June 2025). Posttreatment of raw MS data involved using MZmine 4.1.0 [53], to provide a list of metabolites annotated found in more than 90% of samples. High-confidence annotations [16] were based on MS (*m*/*z* accuracy limit: 5 ppm), MS isotopes/adducts presence, and by matching MS2 data to fragmentation spectra from public libraries (MassBank, GNPS, HMDB, Metabolite Library, MoNA) and/or matching retention times (∆ retention time error: 0.5 min) to those of internal library standards acquired under the same LC-MS conditions. Each metabolite was semi-quantified by its ionic intensity measured by the peak height in each sample. For each feature, details of annotations, metabolite identification confidence, and semi-quantifications in each sample are shown in DataSet S1.

### 4.4. Statistical Analysis

To identify factors affecting carotid atherosclerotic plaque stability, we compared asymptomatic and symptomatic CAS patients according to their clinical and biological characteristics and macroscopic description and molecular composition of their plaque removed by CAE. No prior data were available to support a reliable sample size calculation. For clinical data, statistical analyses involved the Wilcoxon–Mann–Whitney test for continuous data presented as median (interquartile range) and chi-squared test for binary variables. Associations between each annotated metabolite and plaque stability were assessed by adjusted *β*-coefficient (*aβ*) using logistic regression adjusted for North American Symptomatic Carotid Endarterectomy Trial (NASCET) evaluation of CAS and renal function (Modification of Diet in Renal Disease-estimated glomerular filtration rate [eGFR]). Beforehand, logarithmic transformation (base 10) and data scaling (range: 1 to 2) were applied to metabolomic data. Each explanatory feature was presented with its adjusted *β*-coefficient (95% CI) and was considered significant at *p* < 0.05. To evaluate the impact of metabolites on plaque stability, we used a linear Support Vector Machine (SVM) classification for the most significant adducts associated with each metabolite. Predictive properties of the model were assessed from smooth receiver operating characteristic (ROC) curves produced after 100 cross-validations and a confusion matrix. To interpret the results, metabolites were mapped onto biological pathways or grouped according to their sources, using information from the literature or the Human Metabolism Database (HMDB). In addition, correlations between significant metabolites were evaluated by using Spearman coefficients (ρ). MATLAB vR2024a Update 5 was used for statistical analyses.

## 5. Conclusions

In addition to observational data based on dietary habits, we provide molecular-level evidence of the effect of the food exposome on carotid plaque stability. These findings support the beneficial role of plant-based foods, including coffee, in reducing vascular risk. They also reinforce new evidence for the detrimental effects of excessive niacin intake, which may reflect high consumption of processed foods. Plaque stability seems to depend on local and systemic inflammation as well as lipid profiles, highlighting the often-overlooked protective role of HDLc. Metabolomics can pave the way for future multi-omics approaches, enhancing our understanding of how the exposome affects vascular risk.

## Figures and Tables

**Table 1:** Characteristics of patients with symptomatic or asymptomatic carotid atherosclerotic stenosis (CAS) who underwent carotid endarterectomy (CEA).

	All Patients	Asymptomatic Patients	Symptomatic Patients	*p* Value
	*n* = 72	*n* = 42	*n* = 30	
**Medical history before hospitalization**				
Age, y	69.00 [63.75–77.00]	70.00 [64.00–77.50]	69.00 [63.25–76.75]	0.833
Men	56/72 (77.78%)	31/42 (73.81%)	25/30 (83.33%)	0.338
High blood pressure	53/72 (73.61%)	32/42 (76.19%)	21/30 (70.00%)	0.557
Active smoker	43/72 (59.72%)	25/42 (59.52%)	18/30 (60.00%)	0.968
Diabetes mellitus	26/72 (37.50%)	19/42 (45.24%)	8/30 (26.67%)	0.109
Coronary disease	22/72 (30.56%)	15/42 (35.71%)	7/30 (23.33%)	0.261
BMI, kg/m^2^	25.61 [24.09–27.51] (3)	25.48 [24.53–27.72]	26.17 [23.78–27.22] (3)	0.980
Obesity (BMI ≥ 30 kg/m^2^)	12/69 (17.39%)	9/42 (21.43%)	3/27 (11.11%)	0.270
Ischemic stroke or TIA	12/72 (16.67%)	5/42 (11.90%)	7/30 (23.33%)	0.201
**Medical treatment before hospitalization**				
Antiplatelet therapy	53/72 (73.61%)	36/42 (85.71%)	17/30 (56.67%)	0.006
Lipid-lowering therapy	43/72 (59.72%)	32/42 (76.19%)	11/30 (36.67%)	<0.001
Antidiabetes therapy	23/72 (31.94%)	16/42 (38.10%)	7/30 (23.33%)	0.185
Antihypertensive therapy	55/72 (76.39%)	33/42 (78.57%)	22/30 (73.33%)	0.606
**CAS and CEA characteristics**				
NASCET measure of CAS, %	77.50 [70.00–90.00]	77.50 [70.00–90.00]	77.50 [70.00–90.00]	0.939
CEA under antiplatelet therapy	67/72 (93.06%)	39/42 (92.90%)	28/30 (93.30%)	0.938
**Laboratory results at admission**				
Hemoglobin, g/dL	13.55 [12.90–14.90]	13.70 [12.93–14.98]	13.35 [12.90–14.60]	0.451
Hematocrit	0.40 [0.38–0.44]	0.40 [0.38–0.45]	0.40 [0.37–0.43]	0.457
Platelet count, G/L	233.00 [209.00–291.75]	231.50 [203.75–290.00]	240.50 [214.25–288.25]	0.421
Leukocyte count, G/L	8.10 [6.59–9.30] (1)	8.30 [6.95–9.45]	7.92 [6.28–9.10] (1)	0.261
C-reactive protein, mg/L	2.40 [1.18–6.50]	1.75 [0.73–4.65]	3.90 [2.03–7.65]	0.022
Fasting glycemia, g/L	1.00 [0.86–1.19] (2)	1.05 [0.87–1.35] (1)	0.97 [0.86–1.12] (1)	0.331
Total cholesterol, g/L	1.58 [1.30–1.87] (6)	1.60 [1.31–1.78] (4)	1.55 [1.24–1.92] (2)	0.979
LDL-cholesterol, g/L	0.79 [0.56–1.04] (5)	0.72 [0.50–0.91] (3)	0.86 [0.66–1.16] (2)	0.090
HDL-cholesterol, g/L	0.43 [0.36–0.49] (5)	0.46 [0.38–0.57] (3)	0.39 [0.35–0.43] (2)	0.010
Triglycerides, g/L	1.23 [0.96–1.71] (5)	1.25 [0.93–1.87] (3)	1.20 [0.98–1.61] (2)	0.809
Ratio of CRP to HDL-cholesterol	5.68 [2.77–14.92] (5)	4.00 [1.43–10.06] (3)	10.46 [5.03–19.49] (2)	0.004
eGRF, mL/min/1.73 m^2^	75.82 [58.25–93.43]	63.72 [51.84–79.27]	91.14 [76.07–98.93]	<0.001
**Macroscopic description of the plaque after CEA**				
Calcified plaque	17/72 (23.61%)	14/42 (33.33%)	3/30 (10.00%)	0.022
Ulcerated plaque	11/72 (15.28%)	2/42 (4.76%)	9/30 (30.00%)	0.003

Data are median [interquartile range] unless otherwise indicated. BMI: body mass index, CRP: C-reactive protein, HDL-cholesterol: high-density lipoprotein cholesterol, LDL-cholesterol: low-density lipoprotein cholesterol, eGFR: estimated glomerular filtration rate by MDRD (Modification in Diet in Renal Disease), NASCET: North American Symptomatic Carotid Endarterectomy Trial, TIA: transient ischemic attack. The number of missing data is in parenthesis.

## Data Availability

The metabolomics data have been deposited in the MetaboLights repository with the study identifier MTBLS12303 (https://www.ebi.ac.uk/metabolights/MTBLS12303; accessed on 6 June 2025).

## Supplementary Materials

The following supporting information can be downloaded at: https://www.mdpi.com/article/10.3390/ijms26147018/s1.

## Abbreviations

The following abbreviations are used in this manuscript:

AUC	Area Under the ROC curve
BMI	Body Mass Index
CRP	C-Reactive Protein
CAS	Carotid Atherosclerotic Stenosis
CEA	Carotid Endarterectomy
DASH	Dietary Approaches to Stop Hypertension (DASH)
ESI	Electrospray Ionization
eGFR	estimated Glomerular Filtration rate by MDRD (Modification in Diet in Renal Disease)
Col	high performance-liquid chromatography column used
HDL-cholesterol	High-Density Lipoprotein cholesterol
HMDB	Human Metabolome Database
HILIC	Hydrophilic Interaction Liquid Chromatography
IS	Ischemic stroke
LC-MS	Liquid Chromatography coupled with tandem Mass Spectrometry
LDL-cholesterol	Low-Density Lipoprotein cholesterol
2PY	N-methyl-2-pyridone-5-carboxamide
4PY	N1-methyl-4-pyridone-3-carboxamide
NASCET	North American Symptomatic Carotid Endarterectomy Trial
Px	Paraxanthine
Pol	Polarity
PAO	Polyamine Oxydase
ROC	Receiver Operating Characteristic
RT	Retention Time
SSAT	Spermidine/Spermine n-Acetyltransferase
SVM	Support Vector Machine
Tb	Theobromine
Tp	Theophylline
TIA	Transient Ischemic Attack
