# Supplementary material for: Impact of Food Exposome on Atherosclerotic Plaque Stability: Metabolomic Insights from Human Carotid Endarterectomy Specimen
Source: Int J Mol Sci. 2025 Jul 21;26(14):7018. doi: 10.3390/ijms26147018 (PMC12295358; doi:10.3390/ijms26147018)
Supplement: Supplementary file 1 [file ijms-26-07018-s001.zip › supplemental material/supplemental material.pdf]

# Supplemental Material

Figure S1: Pictures of analyzed carotid plaques

Figure S2: Detailed annotations of metabolites significantly associated with plaque stability

Figure S3: Network diagram representing Spearman correlation matrix of significant factors associated with stable plaque

Table S1: Spearman correlation matrix of correlation coefficients for factors significantly associated with stable plaque

**Figure S1:** Pictures of analyzed carotid plaques (n=72)

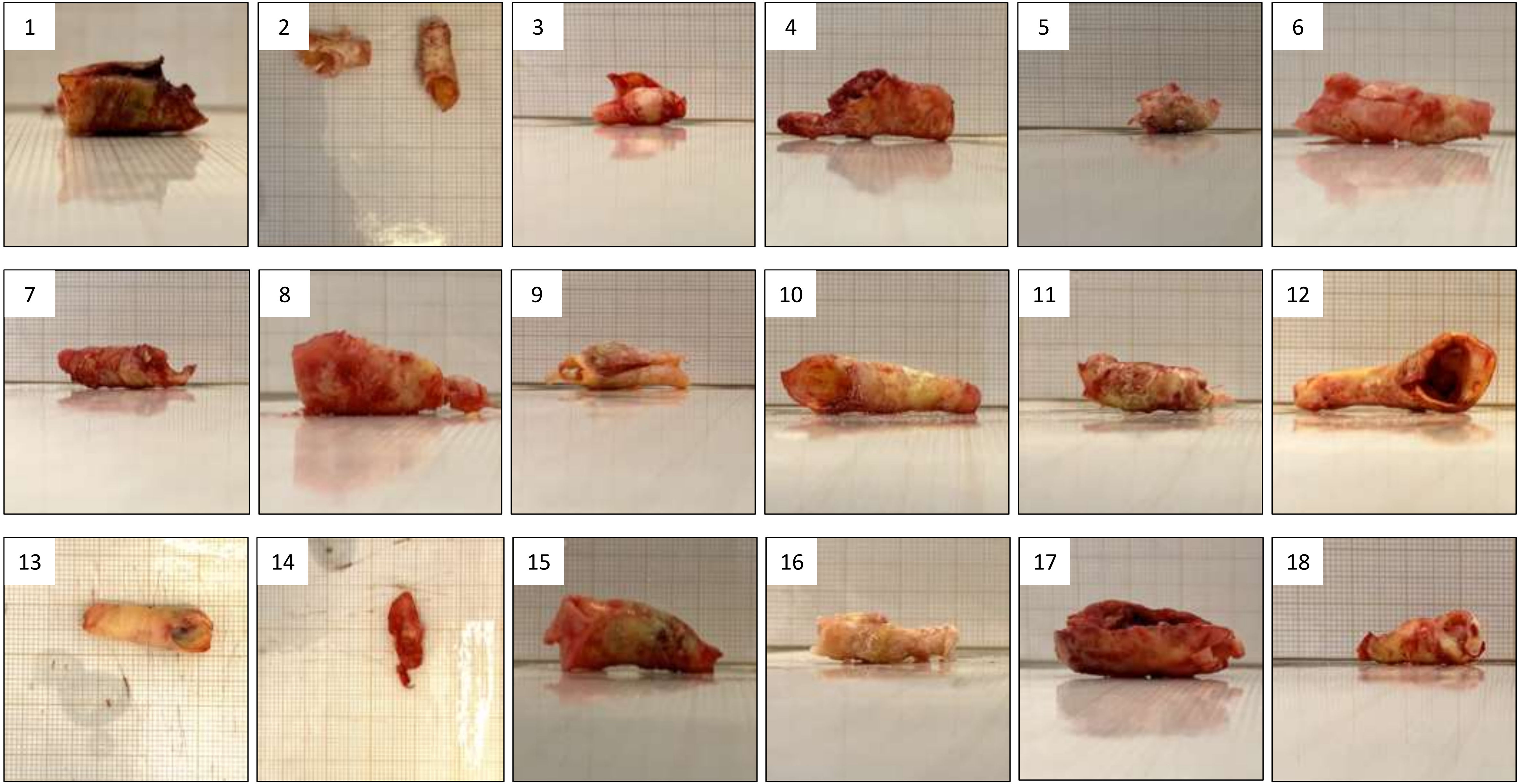
1
2
3
4
5
6
7
8
9
10
11
12
13
14
15
16
17
18

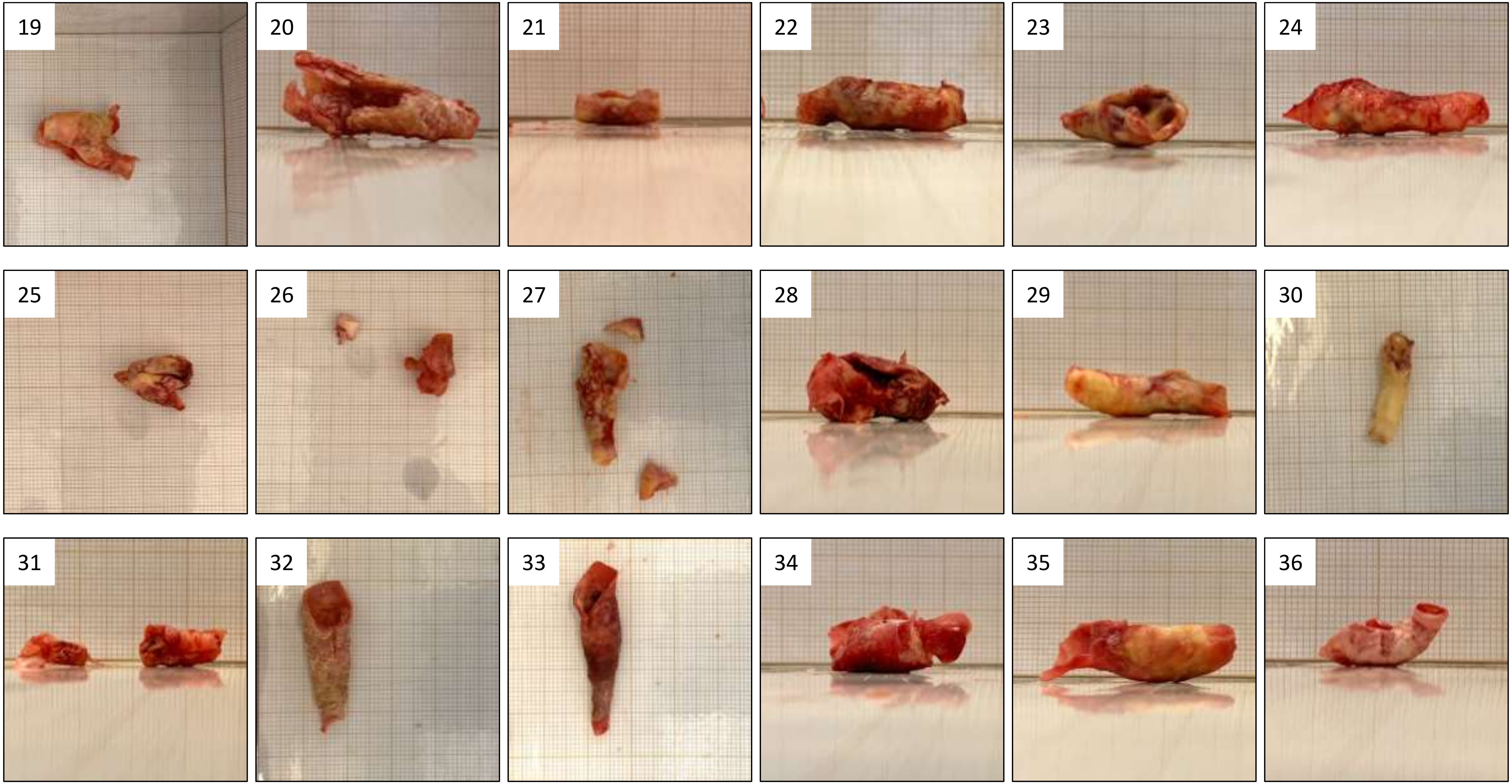
19
20
21
22
23
24
25
26
27
28
29
30
31
32
33
34
35
36

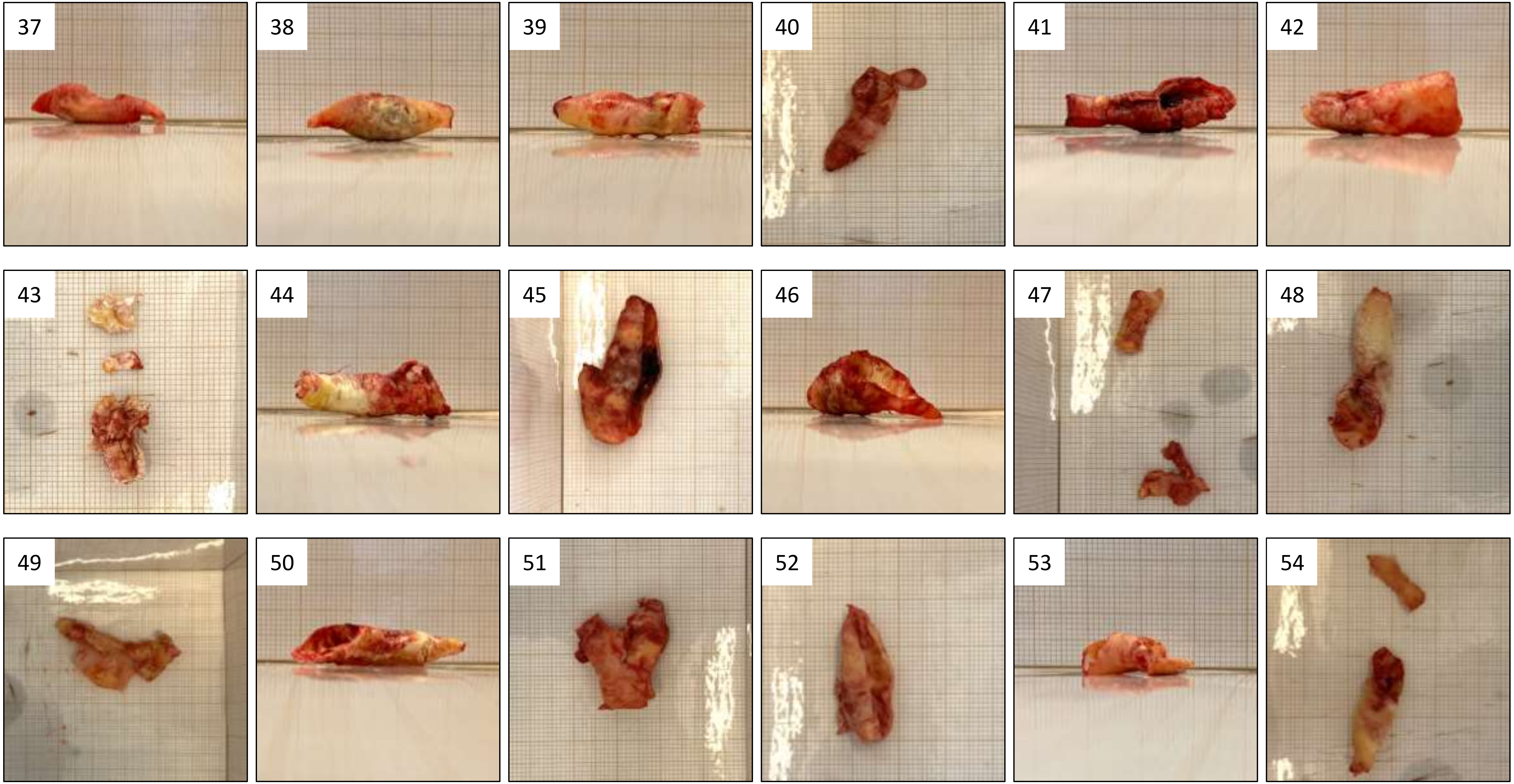
37
38
39
40
41
42
43
44
45
46
47
48
49
50
51
52
53
54

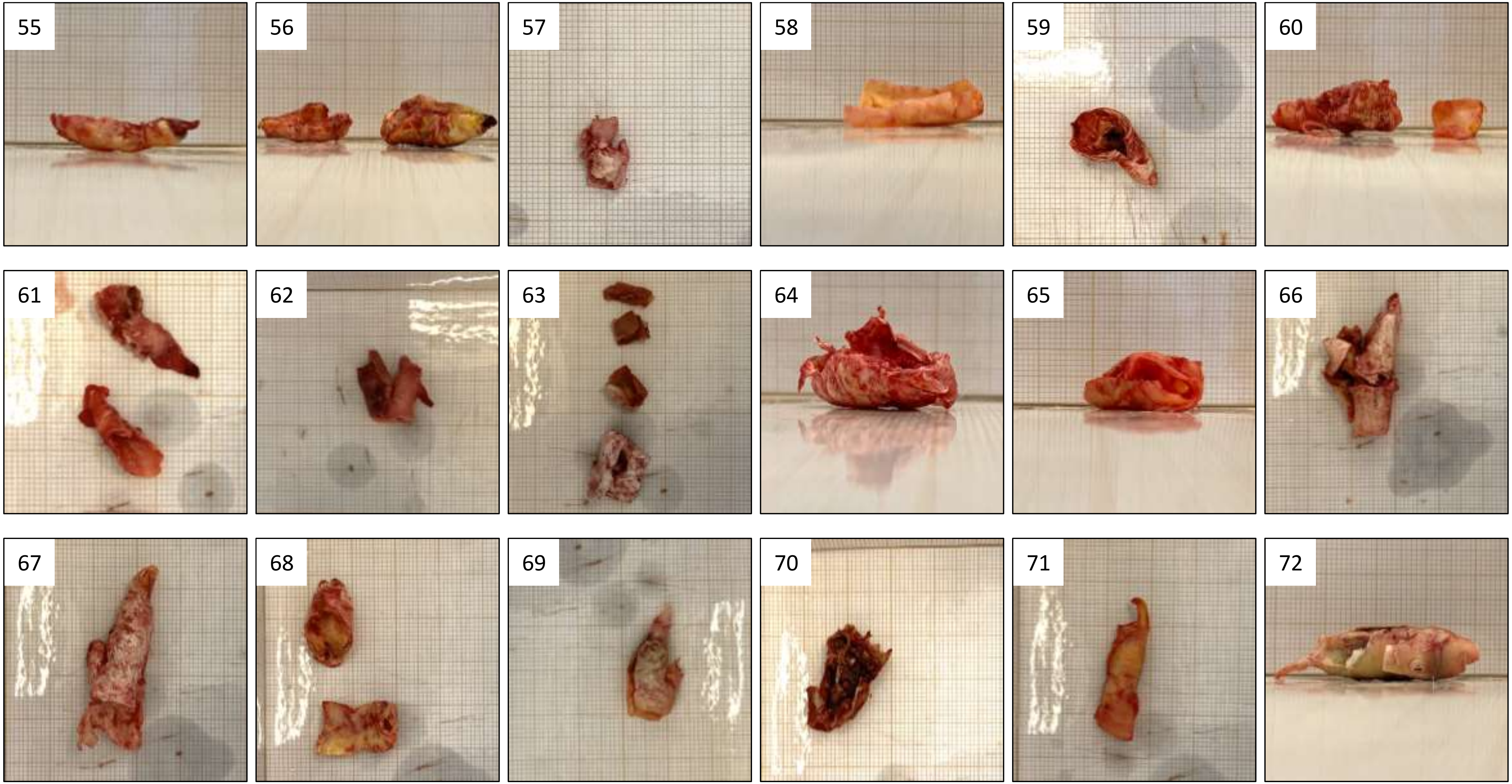
55
56
57
58
59
60
61
62
63
64
65
66
67
68
69
70
71
72

**Figure S2:** Detailed annotations of metabolites significantly associated with plaque stability

| m/z | RT (min) | Column | Polarity | Ion identity | Formula | Theorical mass | Δ mass (ppm) | Isotopes | Theorical RT (min) | Δ RT (%) | Molecule name | HMDB identification number | Shape | MS/MS | Spectral library | Similarity score | Spectral match | Experimental spectrum |
|---|---|---|---|---|---|---|---|---|---|---|---|---|---|---|---|---|---|---|
| 165.0414 | 2.36 | HILIC | ESI- | [M-H]- | C6H6N4O2 | 165.0418 | 2 | yes | 2.23 | 5.84% | 1-Methylxanthine | HMDB0010738 | 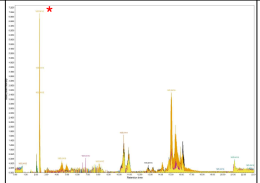 | no | | | | |
| 195.0879 | 1.88 | HILIC | ESI+ | [M+H]+ | C8H10N4O2 | 195.0877 | 1 | yes | 1.87 | 0.58% | Caffeine | HMDB0001847 | 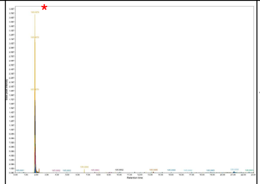 | yes | MASSBANK | 0.911 | 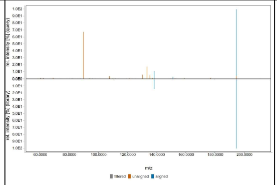 | |
| 195.0878 | 5.07 | C18 | ESI+ | [M+H]+ | C8H10N4O2 | 195.0877 | 1 | yes | 4.85 | 4.44% | Caffeine | HMDB0001847 | 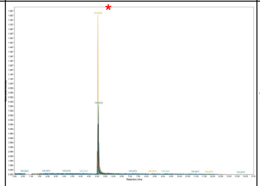 | yes | GNPS | 0.969 | 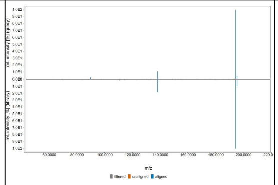 | |
| 153.0191 | 2.23 | HILIC | ESI- | [M-H]- | C7H6O4 | 153.0193 | 1 | yes | 2.18 | 2.37% | Gentisic acid | HMDB0000152 | 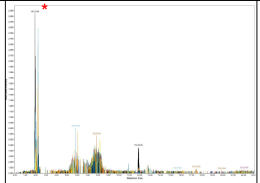 | no | | | | |
| 153.0193 | 5.04 | C18 | ESI- | [M-H]- | C7H6O4 | 153.0193 | 0 | yes | 4.78 | 5.44% | Gentisic acid | HMDB0000152 | 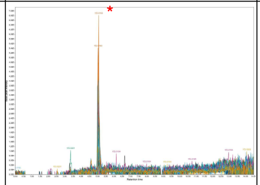 | no | | | | |
| 188.9866 | 3.05 | C18 | ESI- | [M-H]- | C6H6O5S | 188.9863 | 2 | yes | Unknown | Unknown | Hydroquinone sulfate | HMDB0240263 | 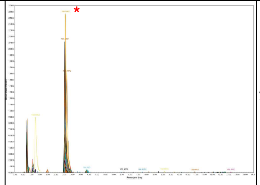 | yes | GNPS | 0.899 | 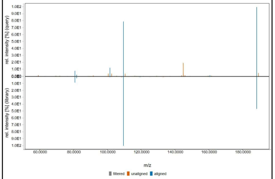 | |

| | | | | | | | | | | | | | | | | |
|---|---|---|---|---|---|---|---|---|---|---|---|---|---|---|---|---|
| 188.9863 | 2.31 | HILIC | ESI- | [M-H]- | C6H6O5S | 188.9863 | 0 | yes | Unknown | Unknown | Hydroquinone sulfate | HMDB0240263 | 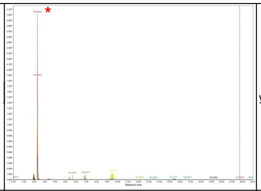 | yes | GNPS | 0.899 | 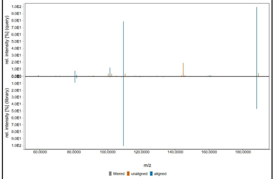 | |
| 175.0480 | 1.67 | C18 | ESI+ | [M+Na]+ | C7H8N2O2 | 175.0478 | 1 | yes | 1.60 | 4.52% | N-methyl-2-pyridone-5-carboxamide (2PY) | HMDB0004193 | 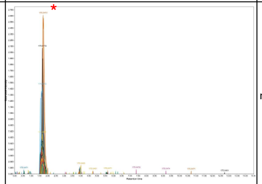 | no | | | | |
| 175.0480 | 1.67 | C18 | ESI+ | [M+Na]+ | C7H8N2O2 | 175.0478 | 1 | yes | Unknown | Unknown | N1-Methyl-4-pyridone-3-carboxamide (4PY) | HMDB0004194 | 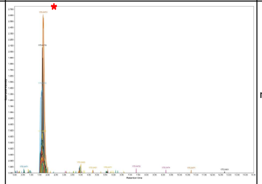 | no | | | | |
| 153.0656 | 1.68 | C18 | ESI+ | [M+H]+ | C7H8N2O2 | 153.0659 | 2 | yes | 1.60 | 4.74% | N-methyl-2-pyridone-5-carboxamide (2PY) | HMDB0004193 | 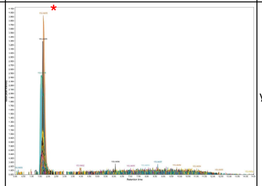 | yes | [25] | Not applicable | 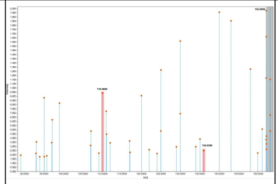 | 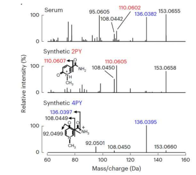  |
| 153.0656 | 1.68 | C18 | ESI+ | [M+H]+ | C7H8N2O2 | 153.0659 | 2 | yes | Unknown | Unknown | N1-Methyl-4-pyridone-3-carboxamide (4PY) | HMDB0004194 | 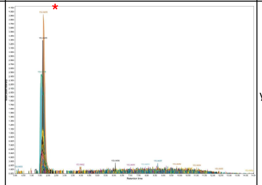 | yes | [25] | Not applicable | 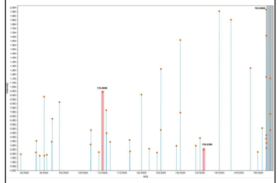 | 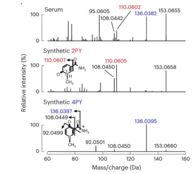  |
| 153.0659 | 2.40 | HILIC | ESI+ | [M+H]+ | C7H8N2O2 | 153.0659 | 0 | yes | 2.21 | 8.66% | N-methyl-2-pyridone-5-carboxamide (2PY) | HMDB0004193 | 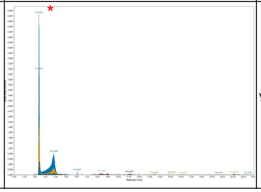 | yes | [25] | Not applicable | 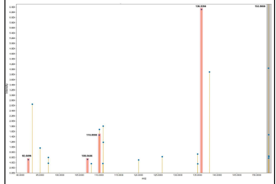 | 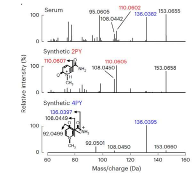  |

| | | | | | | | | | | | | | | | | | | |
|---|---|---|---|---|---|---|---|---|---|---|---|---|---|---|---|---|---|---|
| 153.0659 | 2.40 | HILIC | ESI+ | [M+H]+ | C7H8N2O2 | 153.0659 | 0 | yes | Unknown | Unknown | N1-Methyl-4-pyridone-3-carboxamide (4PY) | HMDB0004194 | 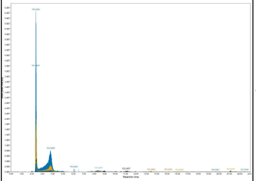 | yes | [25] | Not applicable | 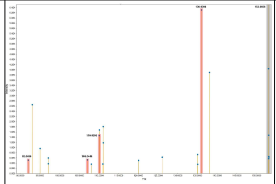 | 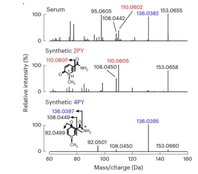 |
| 173.0932 | 0.76 | C18 | ESI- | [M-H]- | C7H14N2O3 | 173.0932 | 0 | yes | 0.73 | 4.11% | N5-Acetylornithine | HMDB0240589 | 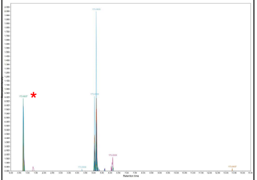 | no | | | | |
| 188.1759 | 17.09 | HILIC | ESI+ | [M+H]+ | C9H21N3O | 188.1757 | 1 | yes | Unknown | Unknown | N8-Acetylspermidine | HMDB0002189 | 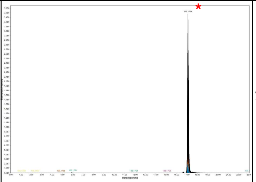 | yes | GNPS | 0.875 | 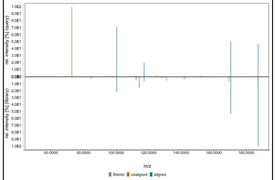 | |
| 210.1580 | 17.10 | HILIC | ESI+ | [M+Na]+ | C9H21N3O | 210.1577 | 1 | yes | Unknown | Unknown | N8-Acetylspermidine | HMDB0002189 | 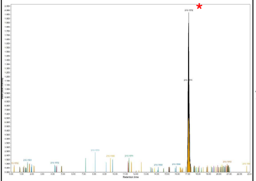 | yes | GNPS | 0.875 | 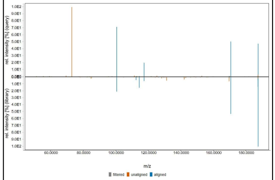 | |
| 188.1757 | 0.64 | C18 | ESI+ | [M+H]+ | C9H21N3O | 188.1757 | 0 | yes | Unknown | Unknown | N8-Acetylspermidine | HMDB0002189 | 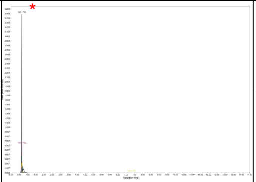 | yes | BIRMINGHAM-UHPLC-MS-POS | 0.855 | 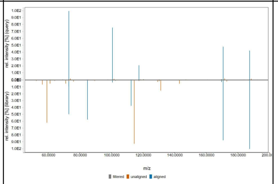 | |
| 179.0569 | 2.24 | HILIC | ESI- | [M-H]- | C7H8N4O2 | 179.0569 | 0 | yes | 2.16 | -3.70% | Paraxanthine | HMDB0001860 | 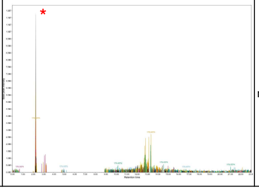 | no | | | | |

| | | | | | | | | | | | | | | | | | | |
|---|---|---|---|---|---|---|---|---|---|---|---|---|---|---|---|---|---|---|
| 179.0569 | 2.24 | HILIC | ESI- | [M-H]- | C7H8N4O2 | 179.0569 | 0 | yes | 2.15 | -4.19% | Theobromine | HMDB0002825 | 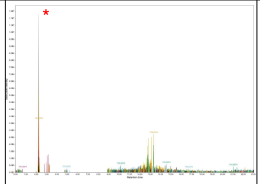 | no | | | | |
| 179.0569 | 2.24 | HILIC | ESI- | [M-H]- | C7H8N4O2 | 179.0569 | 0 | yes | 2.14 | -4.67% | Theophylline | HMDB0001889 | 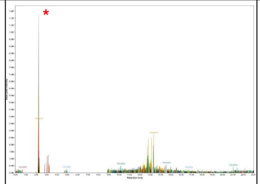 | no | | | | |
| 181.0720 | 2.27 | HILIC | ESI+ | [M+H]+ | C7H8N4O2 | 181.0720 | 0 | yes | 2.15 | 5.59% | Paraxanthine | HMDB0001860 | 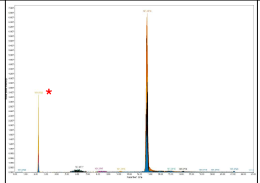 | 0.968 | GNPS | | 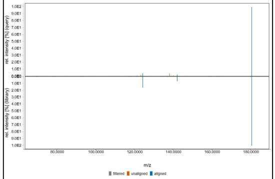 | |
| 181.0720 | 2.27 | HILIC | ESI+ | [M+H]+ | C7H8N4O2 | 181.0720 | 0 | yes | 2.15 | 5.59% | Theobromine | HMDB0002825 | 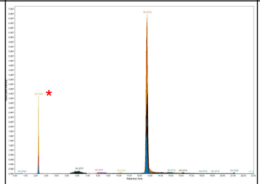 | 0.965 | GNPS | | 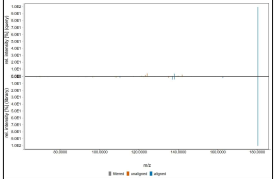 | |
| 181.0720 | 2.27 | HILIC | ESI+ | [M+H]+ | C7H8N4O2 | 181.0720 | 0 | yes | 2.15 | 5.59% | Theophylline | HMDB0001889 | 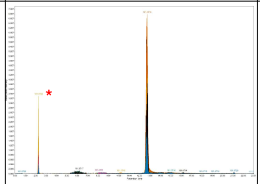 | 0.971 | MASSBANK | | 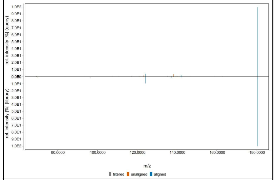 | |
| 181.0720 | 4.56 | C18 | ESI+ | [M+H]+ | C7H8N4O2 | 181.0720 | 0 | yes | 4.41 | 3.43% | Paraxanthine | HMDB0001860 | 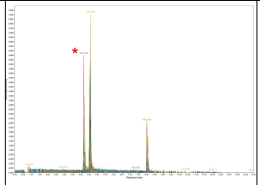 | no | | | | |

| | | | | | | | | | | | | | | | | | | |
|---|---|---|---|---|---|---|---|---|---|---|---|---|---|---|---|---|---|---|
| 181.0720 | 4.56 | C18 | ESI+ | [M+H]+ | C7H8N4O2 | 181.0720 | 0 | yes | 4.41 | 3.43% | Theobromine | HMDB0002825 | 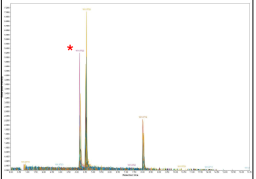 | no | | | | |
| 181.0720 | 4.56 | C18 | ESI+ | [M+H]+ | C7H8N4O2 | 181.0720 | 0 | yes | 4.42 | 3.17% | Theophylline | HMDB0001889 | 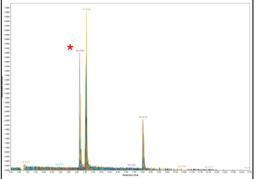 | no | | | | |
| 166.0838 | 0.78 | C18 | ESI+ | [M+Na]+ | C7H13NO2 | 166.0838 | 0 | yes | Unknown | Unknown | Proline betaine | HMDB0004827 | 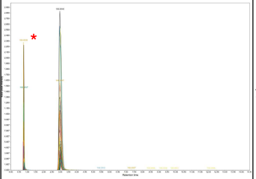 | yes | GNPS | 0.952 | 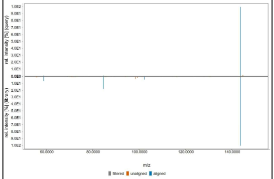 | |
| 158.1177 | 0.86 | C18 | ESI+ | [M+H]+ | C8H15NO2 | 158.1176 | 1 | yes | 0.84 | 2.38% | (S)-Homostachydrine | HMDB0033433 | 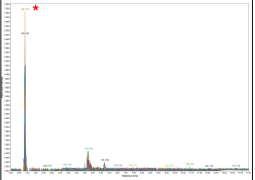 | no | | | | |

**Figure S3:** Network diagram representing Spearman correlation matrix of significant factors associated with stable plaque

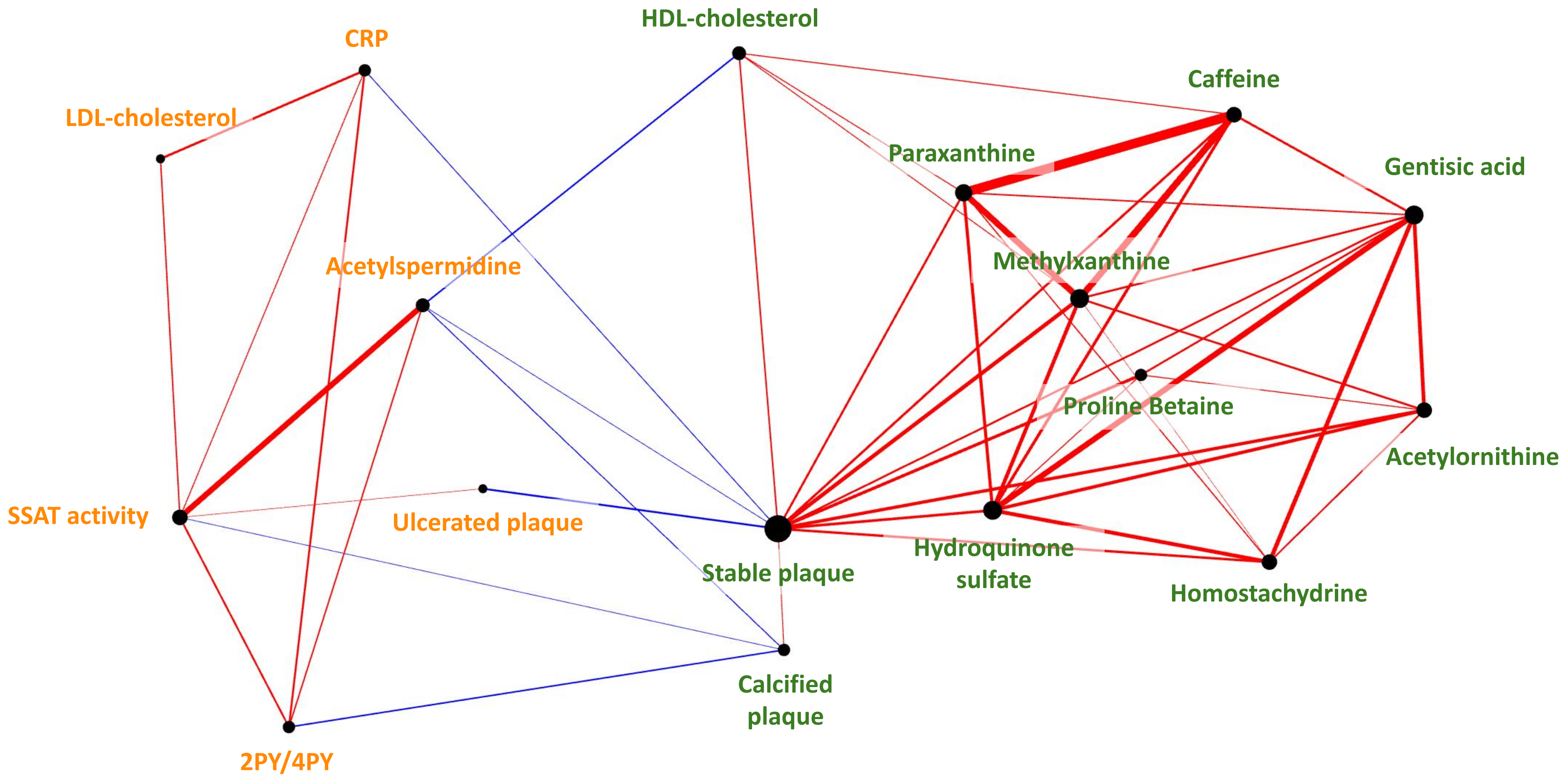


Red: positive correlation; blue: negative correlation; line thickness: strength of correlation. Green factors are associated with stable plaque and orange factors with vulnerable plaque. 2PY/4PY: N-methyl-2-pyridone-5-carboxamide/N1-methyl-4-pyridone-3-carboxamide, SSAT (spermidine/spermine-N1-acetyltransferase) activity assessed by acetylspermidine/spermidine ratio.

**Table S1:** Spearman correlation matrix of correlation coefficients for factors significantly associated with stable plaque. * p<0.05

| | **Stable plaque** | **CRP** | **HDL-cholesterol** | **LDL-cholesterol** | **Calcified plaque** | **Ulcerated plaque** | **Caffeine** | **Paraxanthine** | **Methylxanthine** | **Hydroquinone s.** | **Homostachydrine** | **Gentisic acid** | **Acetylornithine** | **Proline betaine** | **2PY/4PY** | **Acetylspermidine** | **SSAT activity** |
|---|---|---|---|---|---|---|---|---|---|---|---|---|---|---|---|---|---|
| **Stable plaque** | **1.0000** | -0.2726 | 0.3172* | -0.2090 | 0.2709* | -0.3458* | 0.3884* | 0.3687* | 0.5043* | 0.4067* | 0.3877* | 0.3538* | 0.4500* | 0.4392* | -0.1722 | -0.2548* | -0.1749 |
| **CRP** | -0.2726* | **1.0000** | -0.2154 | 0.3797* | -0.0732 | 0.0585 | 0.0052 | -0.0065 | -0.1115 | -0.1710 | -0.2060 | -0.1813 | 0.1218 | -0.0179 | 0.3517* | 0.1406 | 0.2804* |
| **HDL-cholesterol** | 0.3172* | -0.2154 | **1.0000** | -0.0553 | 0.1495 | -0.0740 | 0.2852* | 0.2806* | 0.2772* | 0.0527 | 0.0549 | 0.0401 | 0.0247 | 0.1400 | -0.1716 | -0.3252* | -0.1236 |
| **LDL-cholesterol** | -0.2090 | 0.3797* | -0.0553 | **1.0000** | 0.0598 | -0.0208 | 0.0959 | 0.0459 | -0.0400 | -0.1971 | -0.2318 | -0.0859 | -0.0632 | -0.0825 | 0.1549 | 0.0021 | 0.3067* |
| **Calcified plaque** | 0.2709* | -0.0732 | 0.1495 | 0.0598 | **1.0000** | -0.1452 | 0.1143 | 0.0999 | 0.0417 | -0.0197 | -0.0260 | 0.1849 | 0.0149 | 0.1943 | -0.3171* | -0.2793* | -0.2510* |
| **Ulcerated plaque** | -0.3458* | 0.0585 | -0.0740 | -0.0208 | -0.1452 | **1.0000** | -0.0251 | -0.0306 | -0.1077 | -0.2220 | 0.0288 | -0.1700 | -0.0994 | -0.1328 | 0.1012 | 0.1941 | 0.2443* |
| **Caffeine** | 0.3884* | 0.0052 | 0.2852* | 0.0959 | 0.1143 | -0.0251 | **1.0000** | 0.9277* | 0.7079* | 0.4324* | 0.2006 | 0.3762* | 0.2309 | 0.1439 | 0.0970 | 0.0508 | 0.0399 |
| **Paraxanthine** | 0.3687* | -0.0065 | 0.2806* | 0.0459 | 0.0999 | -0.0306 | 0.9277* | **1.0000** | 0.6739* | 0.4389* | 0.2967* | 0.3130* | 0.1552 | 0.0870 | 0.0165 | 0.1257 | 0.0578 |
| **Methylxanthine** | 0.5043* | -0.1115 | 0.2772* | -0.0400 | 0.0417 | -0.1077 | 0.7079* | 0.6739* | **1.0000** | 0.4906* | 0.2407* | 0.3610* | 0.3589* | 0.2232 | 0.0132 | 0.0724 | 0.0441 |
| **Hydroquinone s.** | 0.4067* | -0.1710 | 0.0527 | -0.1971 | -0.0197 | -0.2220 | 0.4324* | 0.4389* | 0.4906* | **1.0000** | 0.4976* | 0.6412* | 0.4596* | 0.2850* | 0.1074 | 0.0420 | -0.1603 |
| **Homostachydrine** | 0.3877* | -0.2060 | 0.0549 | -0.2318 | -0.0260 | 0.0288 | 0.2006 | 0.2967* | 0.2407* | 0.4976* | **1.0000** | 0.5272* | 0.3321* | 0.2250 | 0.1906 | 0.2635* | 0.0039 |
| **Gentisic acid** | 0.3538* | -0.1813 | 0.0401 | -0.0859 | 0.1849 | -0.1700 | 0.3762* | 0.3130* | 0.3610* | 0.6412* | 0.5272* | **1.0000** | 0.4872* | 0.3510* | 0.1968 | 0.0317 | -0.2213 |
| **Acetylornithine** | 0.4500* | 0.1218 | 0.0247 | -0.0632 | 0.0149 | -0.0994 | 0.2309 | 0.1552 | 0.3589* | 0.4596* | 0.3321* | 0.4872* | **1.0000** | 0.2806* | 0.1988 | -0.0154 | 0.0158 |
| **Proline betaine** | 0.4392* | -0.0179 | 0.1400 | -0.0825 | 0.1943 | -0.1328 | 0.1439 | 0.0870 | 0.2232 | 0.2850* | 0.2250 | 0.3510* | 0.2806* | **1.0000** | -0.0469 | -0.1423 | -0.0434 |
| **2PY/4PY** | -0.1722 | 0.3517* | -0.1716 | 0.1549 | -0.3171* | 0.1012 | 0.0970 | 0.0165 | 0.0132 | 0.1074 | 0.1906 | 0.1968 | 0.1988 | -0.0469 | **1.0000** | 0.3098* | 0.3440* |
| **Acetylspermidine** | -0.2548* | 0.1406 | *-0.3252 | 0.0021 | -0.2793* | 0.1941 | 0.0508 | 0.1257 | 0.0724 | 0.0420 | 0.2635* | 0.0317 | -0.0154 | -0.1423 | 0.3098* | **1.0000** | 0.6390* |
| **SSAT activity** | -0.1749 | 0.2804* | -0.1236 | 0.3067* | -0.2510* | 0.2443* | 0.0399 | 0.0578 | 0.0441 | -0.1603 | 0.0039 | -0.2213 | 0.0158 | -0.0434 | 0.3440* | 0.6390* | **1.0000** |

CRP: C-reactive protein, HDL: high-density lipoprotein, LDL: low-density lipoprotein, 2PY/4PY: N-methyl-2-pyridone-5-carboxamide/N1-methyl-4-pyridone-3-carboxamide, SSAT: spermidine/spermine N-acetyltransferase
